# Supplementary material for: Global temperature modes shed light on the Holocene temperature conundrum
Source: Nat Commun. 2020 Sep 18;11:4726. doi: 10.1038/s41467-020-18478-6 (PMC7501867; doi:10.1038/s41467-020-18478-6)
Supplement: Supplementary file 1 — Supplementary Information [file 41467_2020_18478_MOESM1_ESM.pdf]

Supplementary information for the manuscript 'Global temperature modes shed light on the Holocene temperature conundrum'

Jürgen Bader[1,2], Johann Jungclaus[1], Natalie Krivova[3], Stephan Lorenz[1], Amanda Maycock[4], Thomas Raddatz[1], Hauke Schmidt[1], Matthew Toohey[5], Chi-Ju Wu[3], Martin Claussen[1,6]

[1] Max Planck Institut für Meteorologie, Hamburg, Germany

[2] Uni Climate, Uni Research & the Bjerknes Centre for Climate Research, Bergen, Norway

[3] Max Planck Institut für Sonnensystemforschung, Göttingen, Germany

[4] School of Earth and Environment, University of Leeds, Leeds, UK

[5] GEOMAR Helmholtz Centre for Ocean Research, Kiel, Germany

[6] Centrum für Erdsystemforschung und Nachhaltigkeit (CEN), Universität Hamburg, Hamburg, Germany

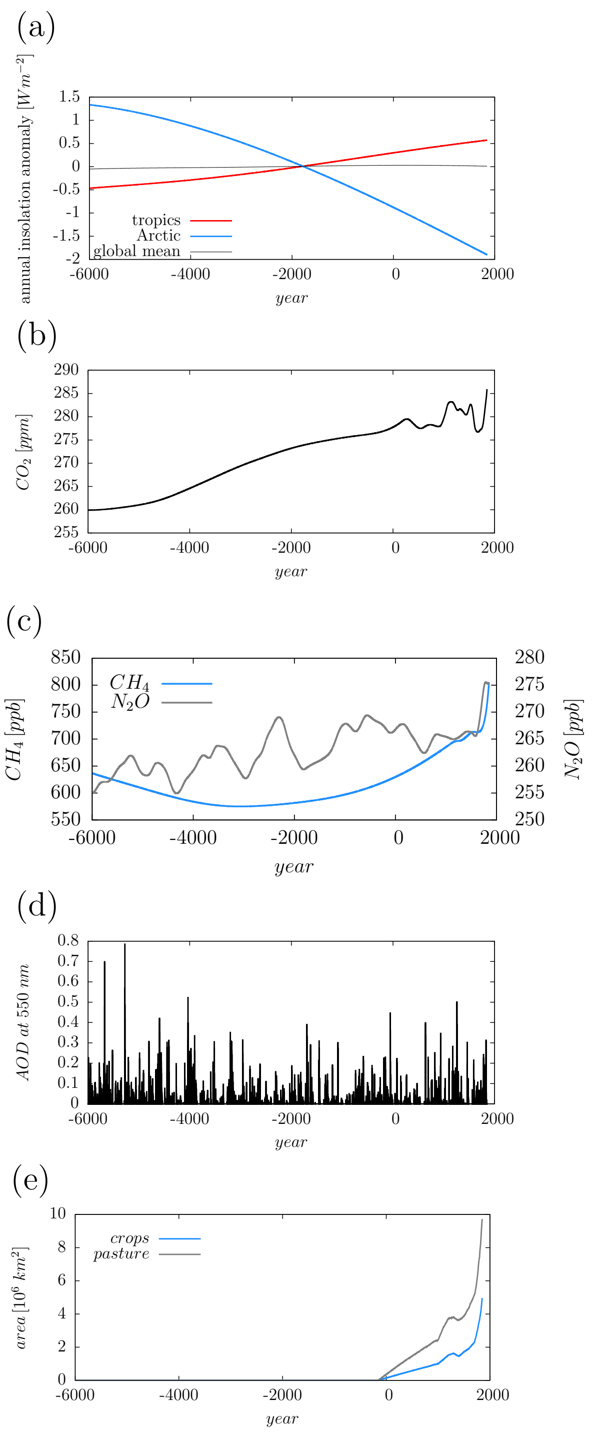

Supplementary Figure 1: **Forcing factors of the transient Holocene simulation.** (Continued on the following page.)

Supplementary Figure 1: (a) Annual incoming solar radiation (insolation) anomaly at the top of the atmosphere. Black curve shows global average, the red curve shows the average over the tropics (30°S to 30°N), and the blue curve shows the average over the Arctic (poleward of 60°N) [Wm^{-2}]. (b) Prescribed atmospheric CO_2 concentration [ppm]. (c) Prescribed atmospheric methane (CH_4) and nitrous oxide (N_2O) concentrations [ppb]. (d) Prescribed Aerosol Optical Depth (AOD) at 550 nm. (e) Prescribed land-use change; globally averaged area for crops and pasture [10^6 km^2].

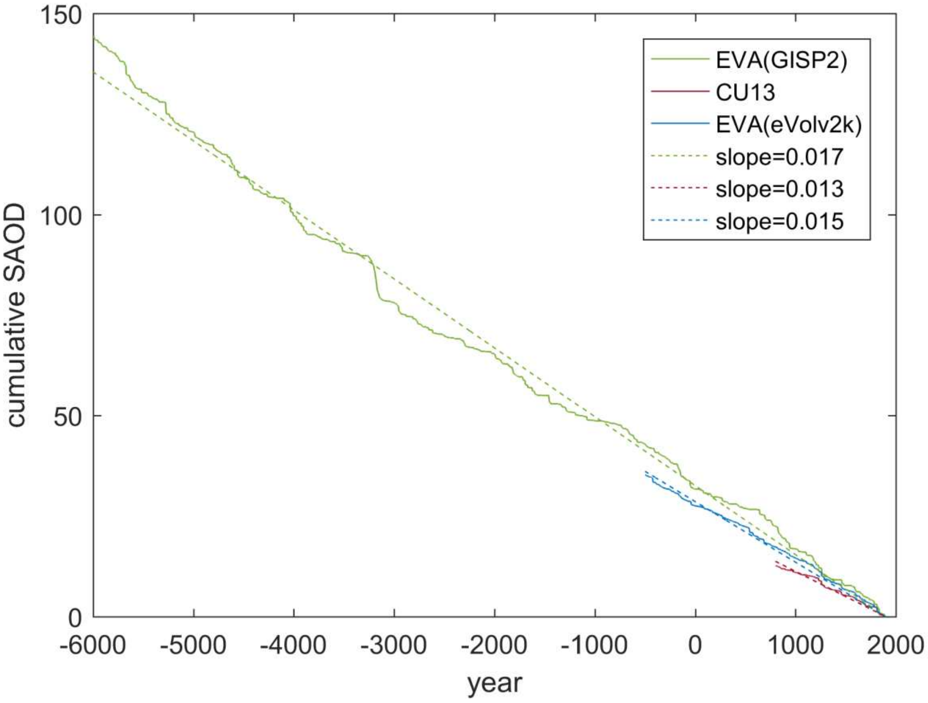

Supplementary Figure 2: **Cumulative global mean stratospheric aerosol optical depth (SAOD)** from the EVA(GISP2) Holocene reconstruction of this work, compared to the Crowley and Unterman[30] (CU13, 850-2000 CE) and EVA(eVolv2k) reconstruction[33] (500 BCE-1900 CE). Annual mean SAOD is summed, counting backwards from the year 1900 for each reconstruction. Linear fits to the time series of cumulative SAOD are performed for each reconstruction, with slopes quoted as positive values.

(a)

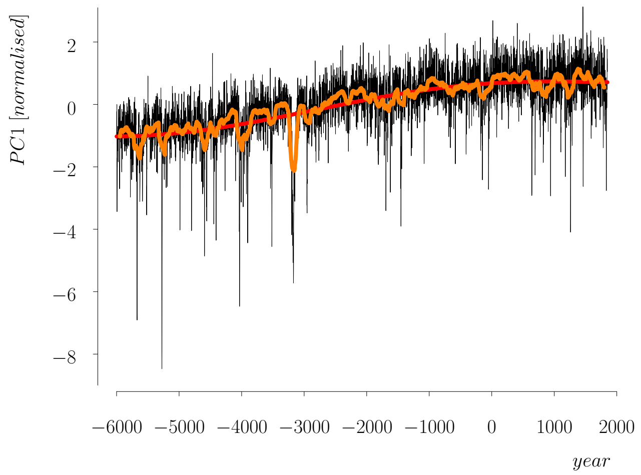

(b)

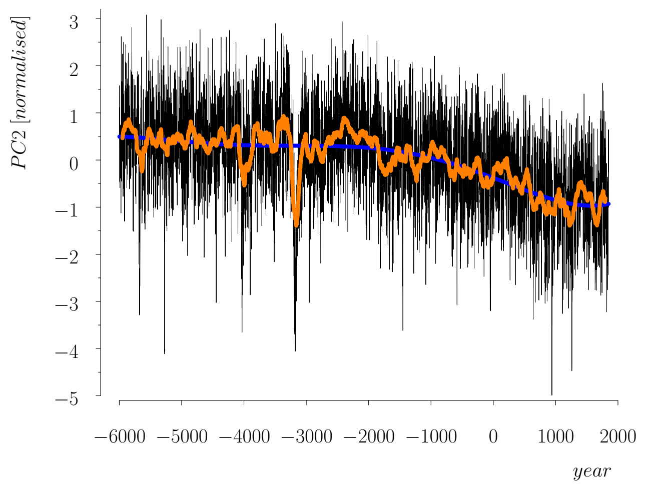

Supplementary Figure 3: **Time series of the EOF analysis.** Normalised unfiltered (black curves) first (a) and second (b) PC based on the simulated annual 2-m temperature using the MPI-ESM data. The red, orange and blue curves show the smoothed PCs by applying either a polynomial fit (blue and red curves) of degree five so that it is a best fit – in a least-squares sense – for the data or by applying a 101-year running mean (orange curves).

Supplementary Notes 1

Robustness of the cooling mode. We have redone the EOF analysis using only high latitude (poleward of 60°N) data from the transient MPI-ESM simulation. The warming and cooling mode patterns using global or high latitude data look quite similar for the high latitude region (Supplementary Figure 4). One difference is that the cooling mode becomes the dominant mode using only high latitude data. This shows that the cooling mode pattern is not an artefact of the orthogonality constrain of the EOF analysis using global data. The loadings of the spatial patterns using regional data are a bit higher compared to the global patterns. The corresponding PCs (Supplementary Figure 5) of the first two modes show a similar low-frequency time evolution. The trend of the warming and cooling mode using only high latitude data are weaker compared to the global PCs – but please note that the spatial patterns have higher loadings.

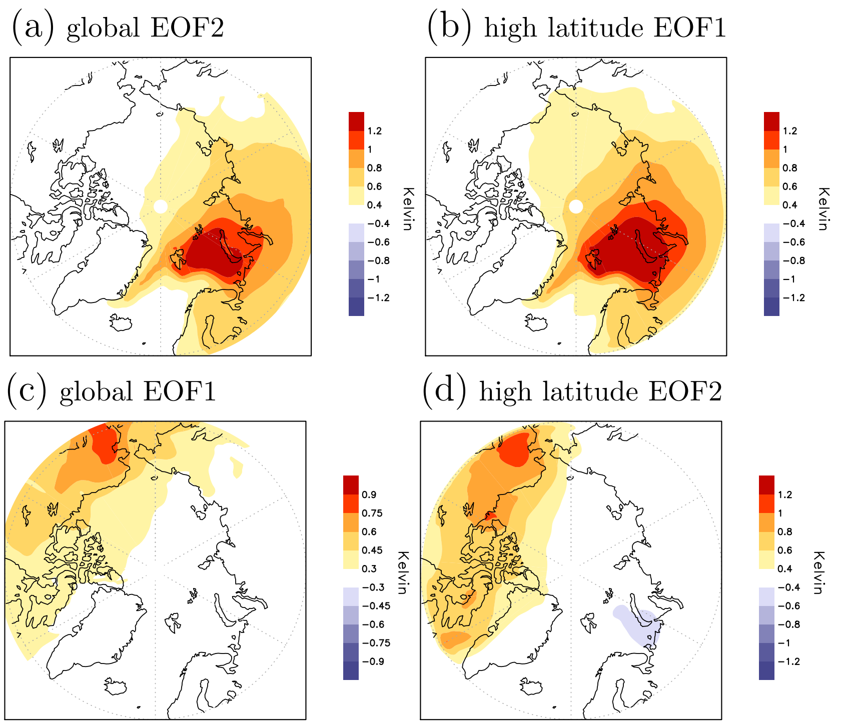

Supplementary Figure 4: **Robustness of the cooling mode.** Comparison between the first two leading EOFs of the annual temperature in the transient MPI-ESM simulation when global data are used (a,c) and when only high latitude (poleward of 60°N) data are used (b,d). The explained variances of the first two regional modes are 30% and 15%.

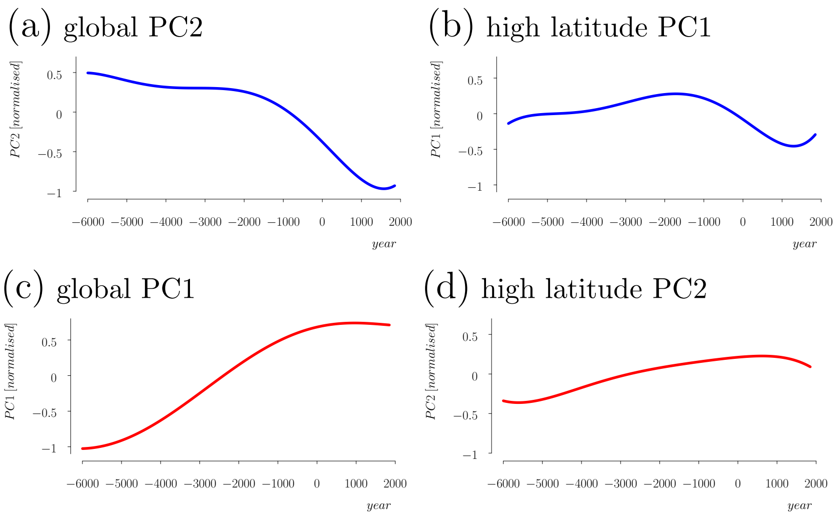

Supplementary Figure 5: **Robustness of the cooling mode.** Comparison between the first two leading PCs of the annual temperature in the transient MPI-ESM simulation when global data are used (a,c) and when only high latitude (poleward of 60°N) data are used (b,d).

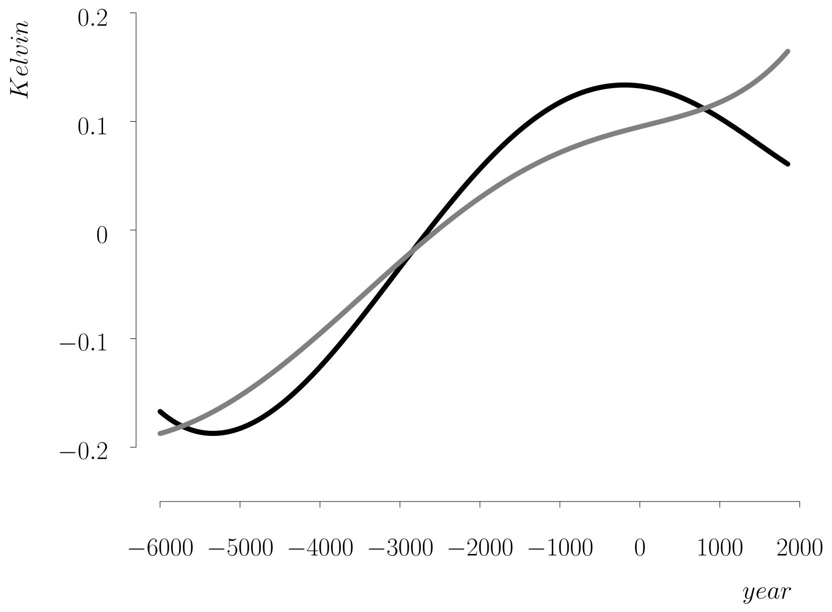

Supplementary Figure 6: **Role of high-frequency forcing.** Annual global mean near-surface temperature anomaly of the transient Holocene simulation using MPI-ESM driven with the full forcing (black curve) and without volcanic forcing and changes in the solar irradiance (grey curve). Both curves show the polynomial fit.

We have performed a similar transient Holocene simulation as described in the main text, except that the external forcing excluded the volcanic forcing and the change in the solar irradience.

Supplementary Notes 2

Additional transient Holocene simulations using different climate models. In addition to the MPI-ESM transient Holocene simulation we have also analysed the “Simulation of the Transient Climate of the Last 21,000 Years (TraCE-21ka)” using CCSM3 climate model and a simulation of the “Quantifying and Understanding the Earth System (QUEST)” project using the FAMOUS model. Please visit the following links for more detailed information regarding the simulations and for accessing the data:

- “https://nerc.ukri.org/research/funded/programmes/quest/”
- “http://www.cgd.ucar.edu/ccr/TraCE/”.

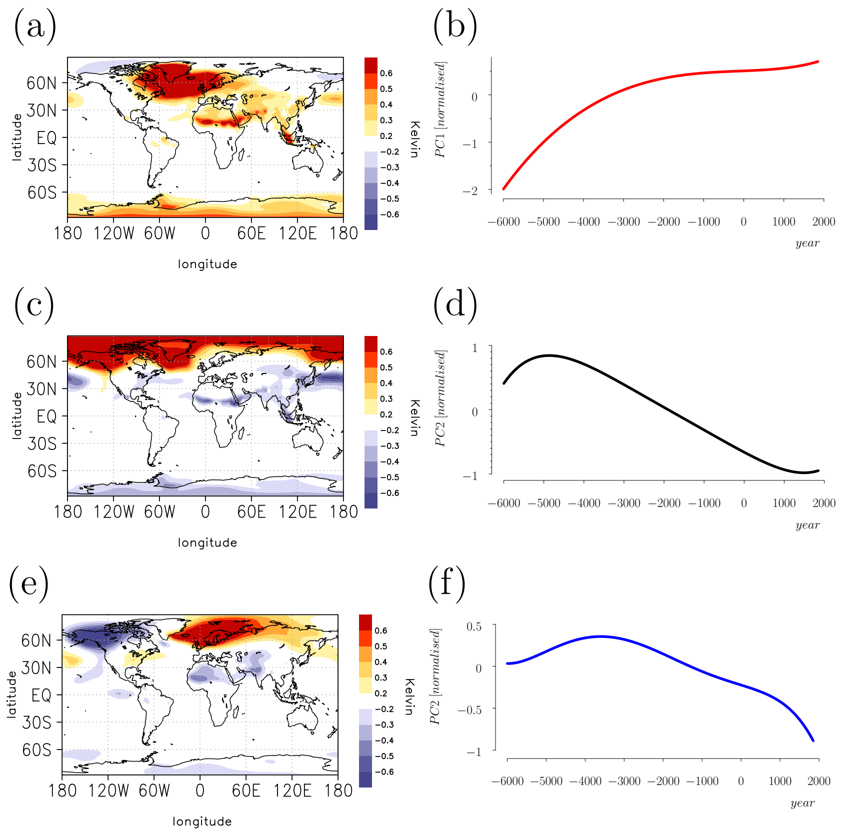

Supplementary Figure 7: **The TraCe simulation.** First three global spatial EOF modes (a,c,e) and corresponding normalised smoothed PCs (b,d,f) based on the simulated annual near-surface temperature using the TraCe data (CCSM3 model). The explained variances of the annual temperature modes are: 17%, 12%, 8%.

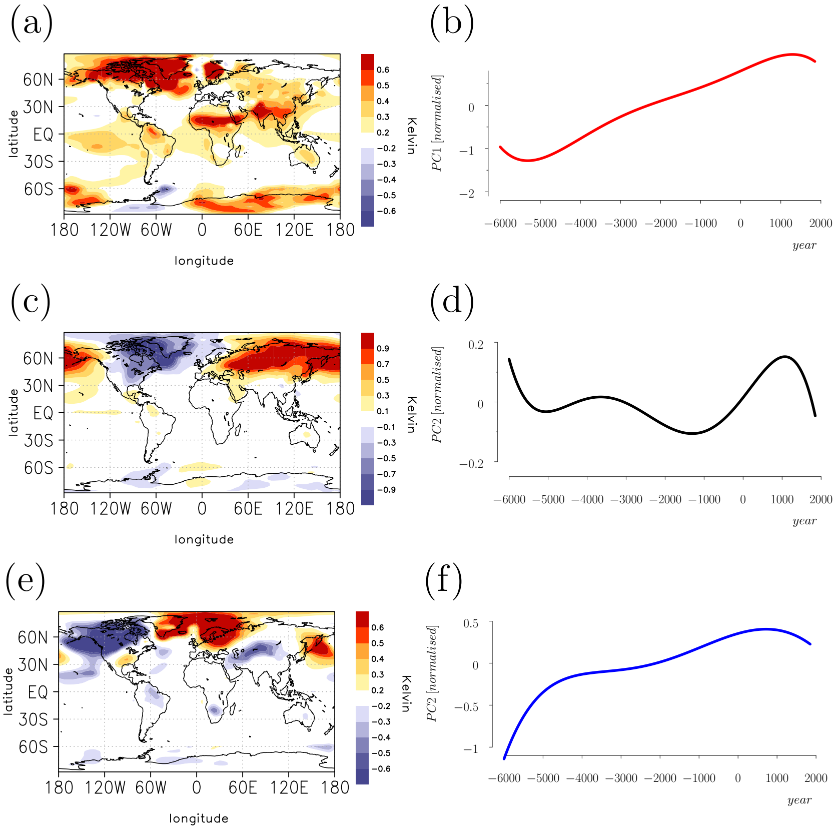

Supplementary Figure 8: **The FAMOUS simulation.** First three global EOF modes (a,c,e) and corresponding normalised smoothed PCs (b,d,f) based on the simulated near-surface temperature using the Quest data (FAMOUS model). The explained variances of the annual temperature modes are: 6%, 6%, 4%.
